# Alterations of host cell ubiquitination machinery by pathogenic bacteria

**DOI:** 10.3389/fcimb.2015.00017

**Published:** 2015-02-27

**Authors:** Jaafar Alomairi, Thomas Bonacci, Eric Ghigo, Philippe Soubeyran

**Affiliations:** ^1^Cellular Stress, Centre de Recherche en Carcérologie de Marseille, INSERM UMR 1068, CNRS UMR 7258, Aix-Marseille University and Institut Paoli-CalmettesMarseille, France; ^2^Infections, Gender and Pregnancy Laboratory, URMITE-IRD198, INSERM U1095, CNRS UMR7278, Aix-Marseille UniversityMarseille, France

**Keywords:** post-translational modifications, ubiquitin, intracellular bacterial pathogens, cell signaling, phagocytosis, xenophagy, immunological response

## Abstract

Response of immune and non-immune cells to pathogens infections is a very dynamic process. It involves the activation/modulation of many pathways leading to actin remodeling, membrane engulfing, phagocytosis, vesicle trafficking, phagolysosome formation, aiming at the destruction of the intruder. These sophisticated and rapid mechanisms rely on post-translational modifications (PTMs) of key host cells' factors, and bacteria have developed various strategies to manipulate them to favor their survival. Among these important PTMs, ubiquitination has emerged as a major mediator/modulator/regulator of host cells response to infections that pathogens have also learned to use for their own benefit. In this mini-review, we summarize our current knowledge about the normal functions of ubiquitination during host cell infection, and we detail its hijacking by model pathogens to escape clearance and to proliferate.

## Introduction

Host invasion by bacteria initiates an immune response which relies on multiple cell populations and communications between them. This normally results in the clearance of the intruder. However, in the case of pathogenic bacteria, host defenses are challenged with specific attacks on their molecular machineries.

Several pathogenic bacteria use different types of apparatus (secretion systems), and various molecules (such as endotoxins and exotoxins) to modulate host cells processes and responses to infection. The pathogenicity of these bacteria is associated with their capacity to survive and replicate within a specialized vacuole or within the cytoplasm of host cells. This can be achieved by avoiding or surviving the phagolysosome formation, escaping the autophagy process of bacteria, a process also known as Xenophagy, and interfering with signaling pathways important for immune response, cell survival, and apoptosis.

Host cells response to invaders depends on the modulation of key cellular functions, from signals transduction to receptors and vesicles trafficking. This rapid tuning is only enabled by post-translational modifications (PTMs) of key proteins implicated in these processes (Broberg and Orth, 2010). These PTMs can be of different kinds, chemical such as protein phosphorylation or peptidic such as protein modification by ubiquitin (ubiquitination) and other ubiquitin-like proteins (Ubls) like SUMOs (Sumoylation) and Nedd8 (Neddylation).

**Figure 1:**
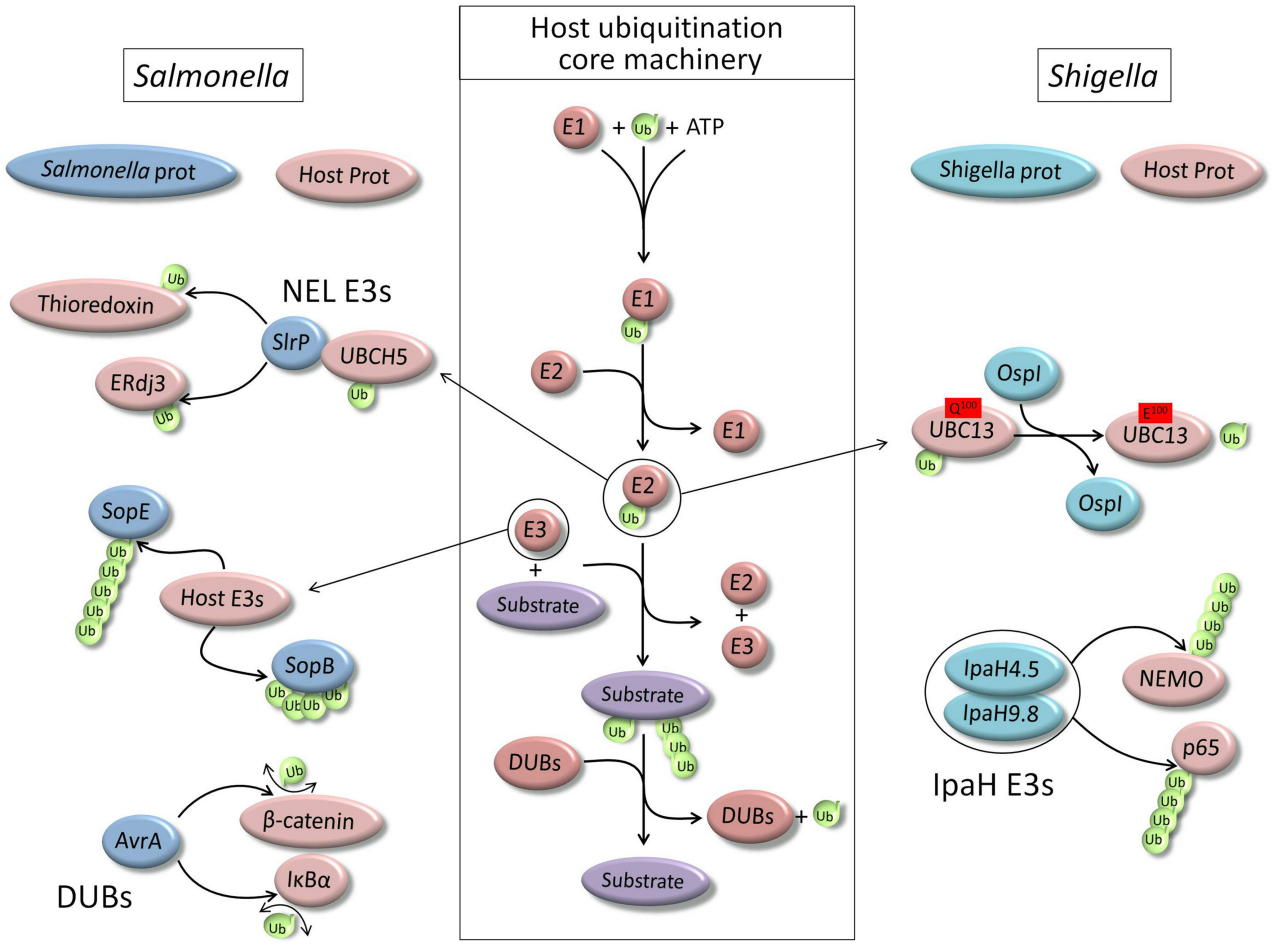
**Schematic representation of the core ubiquitination machinery of the host cell and main examples of its hijacking by two model pathogens, *Salmonella typhimurium* and *Shigella flexneri***.

Ubiquitination is considered as one of the most common PTM, and regulates virtually every intracellular functions as it is involved in essential eukaryotic cellular processes (Hochstrasser, 2009). Ubiquitin is a small 76 amino acids protein which is linked to a lysine residue of the target protein by its carboxyl terminal end to the amino group of the lysine, creating an isopeptide bond. Ubiquitin itself contains seven lysine residues which can be ubiquitinated. This results in the formation of seven different types of polyubiquitin chains, in addition to the linear ubiquitin chain type that consists in the conjugation of one ubiquitin to the N-terminus of another one. PTM by ubiquitin is a three step process requiring the successive action of an activating enzyme (E1), a conjugating enzyme (E2), and a ligase (E3) which gives target specificity (Pickart and Eddins, 2004) (Figure 1). Like any PTM, protein modification by ubiquitin can be reversed by the activity of specific deubiquitinating enzymes (DUBs) (Nijman et al., 2005).

The large variety of regulations mediated by ubiquitin conjugation is also due to this variety of modifications. Indeed, a protein can be mono-ubiquitinated (one ubiquitin on one lysine residue), multi-monoubiquitinated (several mono-ubiquitinated lysine residues), or polyubiquitinated with different kind of polyubiquitin chains (depending on the lysine residue of ubiquitin engaged in the chain). Hence, PTM of proteins by ubiquitin can result in a large variety of modulations, from activity to stability, from interactions to sub-cellular localization. Hence, ubiquitination plays important roles in every crucial step of cellular response to pathogens. Therefore, these kinds of PTMs represent good targets for pathogens to impede host cells defense and to increase their virulence.

## Role of ubiquitin in normal host cell response to non-pathogenic bacteria

When a bacterium is recognized by a host defense cell, such as a macrophage, it is rapidly phagocytosed with the aim to be destroyed. During this process, the bacterium is typically packed into a membrane, forming a phagosome which is addressed to the lysosome. There, membranes from both organelles fused to form the phagolysosome where the acidic pH and degradative enzymes rapidly digest the intruder (15–30 min). PTMs play important roles in every steps of this process and ubiquitination has a particularly important role at the cell signaling level (inflammatory signals) and at the membrane dynamic level (vesicle trafficking and membrane fusion).

### Ubiquitin in infection signaling

The first necessity for host defense cells is to recognize invading bacteria as targets. This necessary step is endorsed by receptors of the Toll-like family, a type of pattern recognition receptors (PRRs), which recognize pathogen-associated molecular patterns (PAMPs). When engaged and activated, these receptors initiate several signaling pathways, major one being the NF-kB pathway which is involved in cytokines production for immune response and cell survival. Interestingly, activation of this pathway is highly dependent on the proteolytic and non-proteolytic ubiquitination of key proteins (Chen, 2005).

NF-kB is a family of heterodimeric transcription factors that, in absence of stimulation, are bound to inhibitory proteins of kB family (IkB) and thereby sequestrated in the cytoplasm.

Bacteria derived molecules, PAMPs, are recognized by toll-like receptors (TLRs) which trigger signaling cascades inside host immune cells once activated (Kawai and Akira, 2010). Upon recognition of PAMPs by TLRs, the kinase IRAK1 (Interleukin-1 Receptor-Associated Kinase 1) is phosphorylated by IRAK4 kinase, and then associates with TRAF6 (TNF receptor associated factor 6), a member of a family of RING-domain E3 ubiquitin ligases (Deng et al., 2000).

TRAF6 then interacts with an E2 ubiquitin-conjugating complex to polymerize K63-linked polyubiquitin chains on itself and on NEMO (NF-kB essential modulator) (Deng et al., 2000; Chen, 2005). Ubiquitinated TRAF6 recruits TAB2 (via its ubiquitin binding domain) and activates the TAB2-associated kinase TAK1 (Tat-associated kinase 1). TAK1 then phosphorylates the beta subunit of IKK complex, which further phosphorylates the inhibitory IkB component of the NF-kB complex. Ubiquitin-activated TAK1 also phosphorylates and activates MKK kinases, such as MKK6, which in turn activates the JNK and p38 kinases pathways (Wang et al., 2001). Phosphorylated IkB is then polyubiquitinated with K48-linked chains and targeted for proteasomal degradation, while releasing NF-kB to activate the transcription of cytokines and chemokines (Kawai and Akira, 2010).

### Ubiquitin and xenophagy

Autophagy is a mechanism by which cells can isolate part of their content in a double membrane structure to create autophagosomes in order to degrade it via its fusion with lysosome (Mizushima et al., 2011). This includes cytosol, old mitochondria, proteins aggregates, and also intruders such as bacteria. Like every kinds of autophagy, this class of autophagy, termed Xenophagy (digestion of foreign materials), is a process highly dependent on ubiquitin and ubiquitin-like conjugation (Kirkin et al., 2009). Since its first observation 30 years ago (Rikihisa, 1984), xenophagy appeared to be crucial for pathogens elimination (Gomes and Dikic, 2014). Pathogens targeting to autophagy for destruction has now been extensively studied and we currently know that this process depends on the core machinery of autophagy. Ubiquitin seems to correspond to a “eat-me” signal for autophagy pathways, and this is also true for xenophagy (Perrin et al., 2004; Kirkin et al., 2009).

Following internalization, some pathogens can actively modify their vacuolar compartment in order to block its maturation, or even escape from it and replicate within the cytosol. Host cells xenophagy can target pathogens at any steps of this process, whether they are in their intact or damaged vacuole or within the cytoplasm. Indeed, ubiquitination can take place on proteins of the damaged membrane (Birmingham et al., 2006) and/or directly on bacterial proteins (Perrin et al., 2004). This ubiquitination depends on the activation of PRRs as well as others danger receptors which can sense perturbations in host cell homeostasis caused by invading bacteria (Chen and Nunez, 2010).

This ubiquitination enables the recruitment of standard autophagy receptors which then initiate the formation of the phagophore (also termed isolation membrane), to which ATG (autophagy-related) proteins are recruited. These autophagy receptors include p62 (Zheng et al., 2009), nuclear domain 10 protein 52 (NDP52) (Thurston et al., 2009), and optineurin (OPTN) (Wild et al., 2011), neighbor of BRCA1 gene 1 (NBR1), or TANK binding kinase 1 (TBK1) (Watson et al., 2012).

## Hijacking of host cell ubiquitination machinery by pathogenic bacteria

Bacterial pathogens have developed multiple ways for manipulating host cell functions to avoid their elimination. As we could previously see, because ubiquitination is involved in major cell signaling responses to infection as well as xenophagy process, interfering with cell host ubiquitination machinery proved to be an efficient way for the survival of many pathogens. Indeed, protein ubiquitination plays a role in any of these processes and pathogens have learned to use it for their own benefit and there are many examples of pathogens interfering with ubiquitination of the host cell. Many pathogenic bacteria utilize specialized type III or type IV secretion systems (T3SS or T4SS) to deliver bacterial effectors proteins into host cells, to modify a variety of cellular processes. There are increasing numbers of effectors that infringe on the ubiquitin pathway, acting as substrates for host cell ubiquitination machinery or as ligases that target specific host and/or bacterial proteins (Figure 1).

### Legionella pneumophila

*Legionella* is a Gram-negative intracellular pathogen that is responsible for a severe pneumonia in humans called as Legionnaire's disease. It establishes a niche called the Legionella-Containing Vacuole (LCV), which is permissive for intracellular bacterial propagation. *Legionella* has a type IV secretion system injecting a cocktail of bacterial proteins targeting host cell processes to support bacterial growth, and numbers of these Icm/Dot effectors contain regions with sequence similarity to F-box or U-box domains contained in eukaryotic E3 ligases (Cazalet et al., 2004; de Felipe et al., 2005). Several of these effectors, such as LegAU13/AnkB, LegU1, and LicA, have been shown to interact with components of the Skp-Cullin-F-box (SCF) ubiquitin ligase complex (Price et al., 2009; Ensminger and Isberg, 2010; Lomma et al., 2010). Moreover, the ubiquitin ligase activity has been verified *in vitro* for LegU1, LegAU13/AnkB (Ensminger and Isberg, 2010) as well as for LubX (Legionella U-box protein) (Kubori et al., 2008). Some substrates for these different *Legionella's* ligases have been identified by using standard interactomic techniques such as yeast two hybrid. Hence, LubX was shown to polyubiquitinate the host cell kinase Clk1 (Kubori et al., 2008) and the *Legionella* effector SidH (Kubori et al., 2010). LegU1 was shown to mediate the ubiquitination of the host cell chaperone BAT3 (Ensminger and Isberg, 2010).

Recently, a unique family of ubiquitin ligases has been identified among *Legionella*'s effectors, SidC (substrate of Icm/Dot transporter C) (Hsu et al., 2014). This protein is anchored to the cytoplamic face of the LCV and recruits host endoplasmic reticulum (ER) proteins to this organelle. Structure analysis revealed the presence of a catalytic triad containing a cysteine, a histidine, and an aspartate residue. It has the capacity to catalyze the formation of high-molecular-weight polyubiquitin chains of different types. Its role is essential for phagosomal membrane remodeling by *Legionella* (Hsu et al., 2014).

### Salmonella typhimurium

*Salmonella* is a common cause of gastroenteritis in humans. It has the ability to invade non-phagocytic cells such as enterocytes of the intestinal epithelium. This capacity depends on a T3SS, known as T3SS1. A second T3SS, T3SS2, is required for post-invasion establishment of the replicative niche, a modified phagosome known as the *Salmonella*-containing vacuole (SCV) (Steele-Mortimer, 2008). Several *Salmonella* effectors, from both T3SS1 and T3SS2, alter host cell ubiquitin pathways.

Invasion of host cells by Salmonella depends on the sequential activity of SopE, a guanine nucleotide exchange factor (GEF) which activates Cdc42 and Rac1 (Hardt et al., 1998), and of SptP, a GTPase-activating protein (GAP) which inactivates SopE (Fu and Galan, 1999). Actually, both proteins are targeted for ubiquitin-dependent degradation, but SopE is degraded more efficiently and therefore inactivated more rapidly than SptP (Kubori and Galan, 2003).

SopB, an inositol phosphate phosphatase that has several functions during invasion (Steele-Mortimer et al., 2000; Bakowski et al., 2010), is another essential effector for *Salmonella* virulence. Following delivery into host cells, SopB is monoubiquitinated on at least six lysine residues, via a mechanism that does not require any of the known *Salmonella* E3 ubiquitin ligases (Knodler et al., 2009). This ubiquitination down-regulates SopB activity at the plasma membrane but increases its retention on the SCV. Hence, depending on its ubiquitination status, SopB has several functions, ranging from actin-mediated bacterial internalization and Akt activation to vesicular trafficking and intracellular bacterial replication at the phagosome (Knodler et al., 2009).

Some *Salmonella* effectors are real ubiquitin enzymes acting as ligases or DUBs. Based on functional and structural data, SopA is a novel HECT-like E3 ligase, although it has little sequence similarity with any eukaryotic E3 ligase. SopA was shown to form an Ub-thioester intermediate and its crystal analysis revealed a C-terminal domain architecture that resembles the N- and C-lobe arrangement of HECT domains (Diao et al., 2008). It has been shown to interact with the host cell conjugating enzyme UbcH7 (Lin et al., 2012). But so far, no substrate has been identified. Interestingly, SopA can be targeted for degradation following ubiquitination by the endoplasmic reticulum (ER)-bound RING finger protein 5 (RNF5/RMA1) (Zhang et al., 2005), a protein part of the ER-anchored Ubiquitin ligase complex which processes malfolded proteins (Delaunay et al., 2008).

Three other effectors of *Salmonella*, SlrP, SspH1 and SspH2, are ligases of the NEL family (Novel E3 Ligase). The NEL domain contains a conserved catalytic cysteine residue involved in E2 binding and ubiquitination reaction (Quezada et al., 2009), as well as a leucine-rich repeat (LRR) of variable length supposedly involved in substrate-recognition (Quezada et al., 2009). Whereas SspH2 is injected into host cells only by T3SS2 (Miao et al., 1999), SlrP and SspH1 are translocated via both T3SS1 and T3SS2. Hence SspH2 has function in late infection whereas Slrp and SspH1 play a role during the early steps of infection. The NEL domain of these ligases has no equivalent among all known mammalian ligases but these ligases are very efficient in using the host cells ubiquitination machinery such as the conjugating enzyme UBCH5, and the negative regulation of their activity seems to be realized upon the binding of the LRR to a target protein (Quezada et al., 2009). Only few potential host substrates have been identified, such as PKN1 (protein kinase called protein kinase N 1) for SspH1 (Haraga and Miller, 2006), and Thioredoxin and ERdj3 for SlrP (Bernal-Bayard and Ramos-Morales, 2009; Bernal-Bayard et al., 2010). However, the biological outcome of these identifications still needs further investigation.

At least two *Salmonella* effectors are deubiquitinases, SseL and AvrA, and both were shown to be involved in down regulating immune signaling (Collier-Hyams et al., 2002; Le Negrate et al., 2008). AvrA was supposed to have anti-inflammatory effects because of its ability to deubiquitinate number of proteins, such as Iκ B-α and β-catenin, thereby regulating host inflammatory responses through NF-κ B (Collier-Hyams et al., 2002) and β-catenin (Sun et al., 2004). However, it has also been shown that AvrA has no significant anti-inflammatory function when injected by *Salmonella* at endogenous levels (Du and Galan, 2009). Therefore, the real function of AvrA still needs to be fully determined. Similarly, recent reports showed that SseL has no effect, negative or positive, on the NFkB pathway (Mesquita et al., 2013), but its deubiquitinase activity was shown to reduce the autophagic flux in infected cells and to favor bacterial replication (Mesquita et al., 2012).

Finally, a recent study of the impact of *Salmonella* LPS stimulation on the ubiquitination profile of macrophages revealed a profound and global alteration of this PTM in the host cell (Nakayasu et al., 2013). This change negatively modulates the activity of DUBs, resulting most likely in the polyubiquitination and degradation of specific proteins such as DBC1 (deleted in breast cancer 1), a histone deacetylase (HDAC) inhibitor that controls chromatin remodeling during inflammatory response. This work is a unique example showing that bacterial membrane associated factors can also interfere with many ubiquitination pathways of the host cell.

### Shigella flexneri

*Shigella* is a Gram-negative pathogenic bacterium which causes shigellosis in human by invading intestinal epithelial cells, after it has been ingested. *Shigella* delivers effectors into host cells via a type III secretion system in order to modulate cellular processes and to favor multiplication (Ashida et al., 2011). As usual, several targets of these effectors are signaling pathways important for host defense cell. The phosphothreonine lyase activity of OspF effector inhibits the MAPK signaling pathway by irreversibly dephosphorylating MAPKs, Li et al. (2007) and Zhu et al. (2007). IpaH9.8 and IpaH4.5, which belong to a new IpaH family of E3 ubiquitin ligases (Rohde et al., 2007), inhibit the NF-κ B signaling pathway by mediating the ubiquitination of NEMO and of p65 (Ashida et al., 2010; Wang et al., 2013).

Moreover, the VirA effector of *Shigella* inactivates Rab1 with TBC-like GAP activity, inhibiting the host cell autophagy-mediated defense (Dong et al., 2012).

A recent study revealed that a newly identified *Shigella* effector, OspI, targets the host UBC13 by deamidating glutamine 100, producing a glutamate residue, and leading to the disruption of TRAF6-catalyzed polyubiquitination (Sanada et al., 2012). The disruption of TRAF6 polyubiquitination suppresses the diacylglycerol-CBM (CARD-BCL10-MALT1 complex)-TRAF6-NF-κ B signaling pathway and significantly reduces the host inflammatory responses (Sanada et al., 2012). OspI targets UBC13 via extensive interactions and UBC13 binding remodels the structure of OspI for catalysis. The structural analysis of UBC13 in complex with OspI, TRAF6, CHIP, and OTUB1 revealed that OspI binds to the same surface region on UBC13 as the host proteins (Fu et al., 2013).

OspG is an effector kinase whose function during invasion is to suppress the host inflammatory response. OspG can interact with at least 10 distinct human ubiquitin-charged E2 conjugating enzymes, and this binding strongly enhances the kinase activity of OspG (Pruneda et al., 2014).

### Listeria monocytogenes

*Listeria* is the causative agent of listeriosis, a serious invasive disease that primarily affects pregnant women, newborns and immunocompromised individuals (Bonazzi et al., 2009). It can invade host cells through two different pathways, depending on which cell surface receptors is engaged, internalin A (InlA) which binds to E-cadherin of the host cell, or internalin B (InlB) which binds to c-Met (Braun et al., 1999; Lecuit et al., 1999). Both pathways involve PTMs of host cell proteins, such as ubiquitination, as well as actin remodeling (Cossart and Lecuit, 1998; Bonazzi et al., 2008).

The surface-bound protein InlA binds to E-cadherin, a cell to cell adhesion molecule that forms a physical link between the cell membranes of adjacent cells (Mengaud et al., 1996). In epithelial cells, E-cadherin complexes are endocytosed following activation of the tyrosine kinase Src, which induces tyrosine phosphorylation of E-cadherin thus enabling its subsequent phospho-dependent ubiquitination by the ubiquitin ligase Hakai (Fujita et al., 2002). Internalization of *Listeria* via InlA induces the same phospho-dependent ubiquitination of E-cadherin followed by clathrin-dependent endocytosis (Sousa et al., 2007).

InlB binds to the host cell receptor c-Met, a RTK (Receptor Tyrosine Kinase) (Shen et al., 2000) which is normally activated by HGF (Hepatocyte Growth Factor). InlB interacts with the first immunoglobulin-like domain and the Sema domain of c-Met thereby stabilizing the receptor that can initiate signaling (Niemann et al., 2007). Activation of c-Met receptor leads to its clathrin-dependent internalization and its down-regulation, a process that requires the ubiquitin ligase c-Cbl, which is recruited to c-Met in a phospho-dependent manner (Peschard et al., 2001). Binding of InlB to c-Met induces the c-Cbl-dependent ubiquitination and endocytosis of c-Met and so the internalization of the bacteria. Importantly, *Listeria* invasion is directly dependent on the c-Cbl mediated ubiquitination of the receptor (Veiga and Cossart, 2005).

This bacterium also uses the host cell ubiquitination machinery to target some of its own proteins. Listeriolysin O (LLO), a pore-forming toxin that is essential for *Listeria* to escape from the phagosome into the host cell cytoplasm, may also be deleterious for the pathogen if not tightly regulated. Hence LLO is normally ubiquitinated and degraded by host cell machinery, and stabilizing mutation or overexpression of LLO seriously decreases the virulence of this bacterium (Schnupf et al., 2007).

## Concluding remarks

Pathogenic bacteria have coevolved with their target organisms and therefore they have learned how to use and/or subvert their defense mechanisms. The cell response to bacterial invasion needs to be rapid and hence relies on PTMs of key proteins. Ubiquitination appeared to be one of these PTMs important for host cell defense that is targeted by pathogens. These last years, new tools have been developed to explore PTMs dynamics (Vertegaal, 2011; Bonacci et al., 2014) that will surely help to identify new important mechanisms enabling pathogens to survive and proliferate within host cells.

### Conflict of interest statement

The authors declare that the research was conducted in the absence of any commercial or financial relationships that could be construed as a potential conflict of interest.
